# Supplementary material for: Salmonella Typhimurium and Pseudomonas aeruginosa Respond Differently to the Fe Chelator Deferiprone and to Some Novel Deferiprone Derivatives
Source: Int J Mol Sci. 2021 Sep 23;22(19):10217. doi: 10.3390/ijms221910217 (PMC8508819; doi:10.3390/ijms221910217)
Supplement: Supplementary file 1 [file ijms-22-10217-s001.zip › ijms-1321497-supplementary.pdf]

Supplementary Materials

Table S1. Bacterial strains

Name	Relevant genotype	Source or Ref
Salmonella enterica **ser. Typhimurium**		
ATCC® 14028™	wild type	Lab collection
SA330	*fepA/entF*::kan	[25]
SA213	*iroB*-3Xflag (kan) *ilvI*::Tn10dTac-cat::3Xflag	[26]
MC120	*sodB*-3Xflag (kan)	[25]
Pseudomonas aeruginosa		
PAO1	wild type	Lab collection
pchDpvdA	*pchD pvdA*	[15]
Escherichia coli		
DH5α	Prom-*pchR* pMP220	Lab collection
DH5α	Prom-*pvdS* pMP220	Lab collection
DH5α	Prom-*feoA* pMP220	Lab collection
HB101	pRK2013	Lab collection

Table S2. Structures of DFP - derivative compounds

Name	Structure
**1**	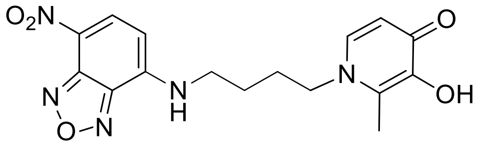
**2a**	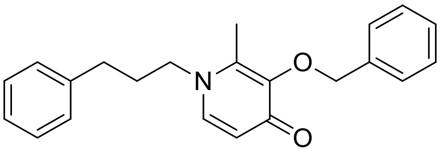
**2b**	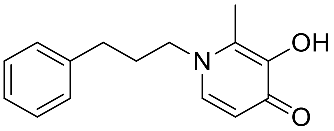
**3a**	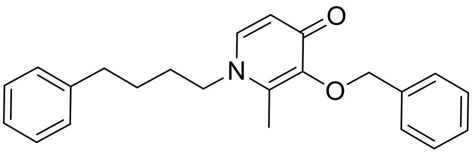
**3b**	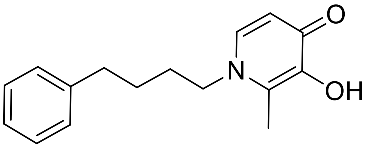
**4a**	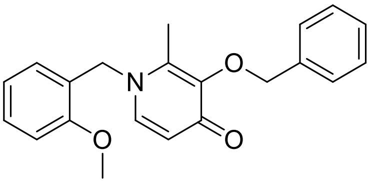
**4b**	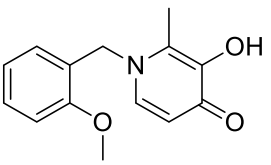
**5a**	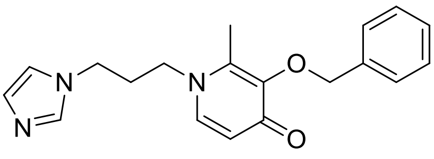

**5b**	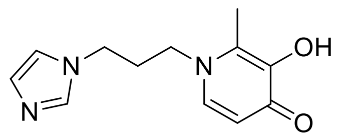
**6a**	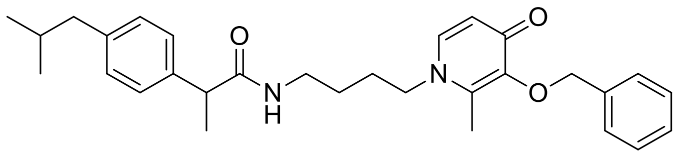
**6b**	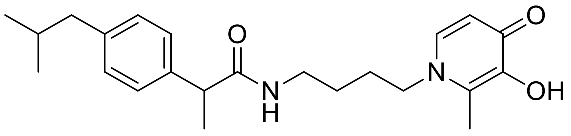
**7a**	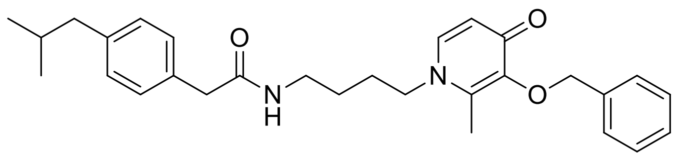
**7b**	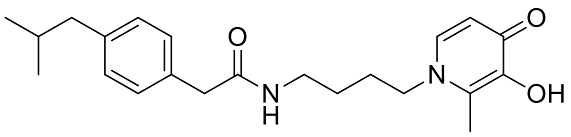
**8a**	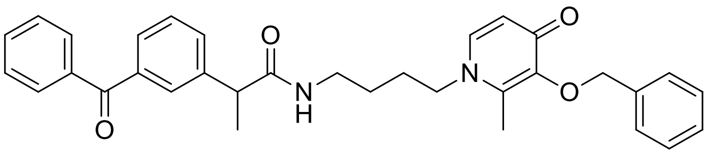
**8b**	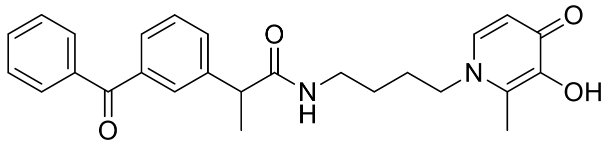

	$A_{407/600}$
PAO1	0.0030 ± 0.0003
PAO1 + DFP 1 mM	0.0385 ± 0.0032

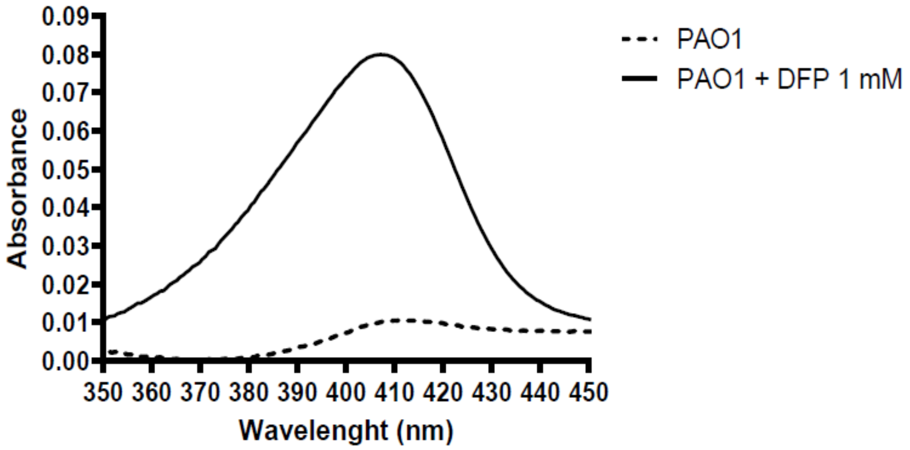

Figure S1: DFP induces pyoverdine production in *P. aeruginosa* cultivated in LB. PAO1 Was grown for 24 h at 37°C in LB in the absence of DFP or in presence of 1 mM DFP. Bacteria were harvested by centrifugation and the supernatant was analyzed to detect the presence of pyoverdine. Both the A_{407}/A_{600} ratio and the spectra of the supernatants indicate the accumulation of a species with and absorption peak centered close to 407 nm (pyoverdine) in bacteria grown in presence of DFP.

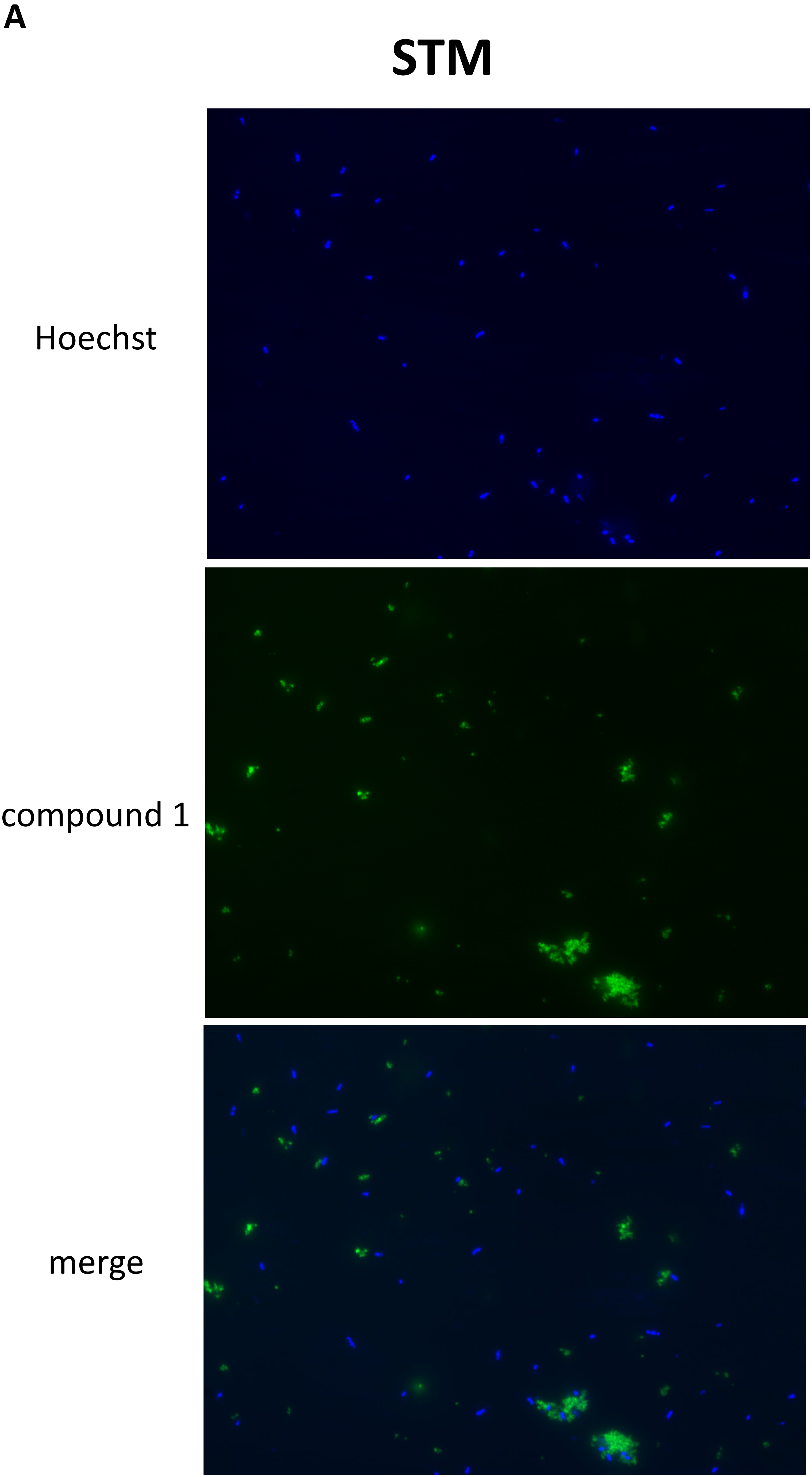
A
STM
Hoechst
compound 1
merge

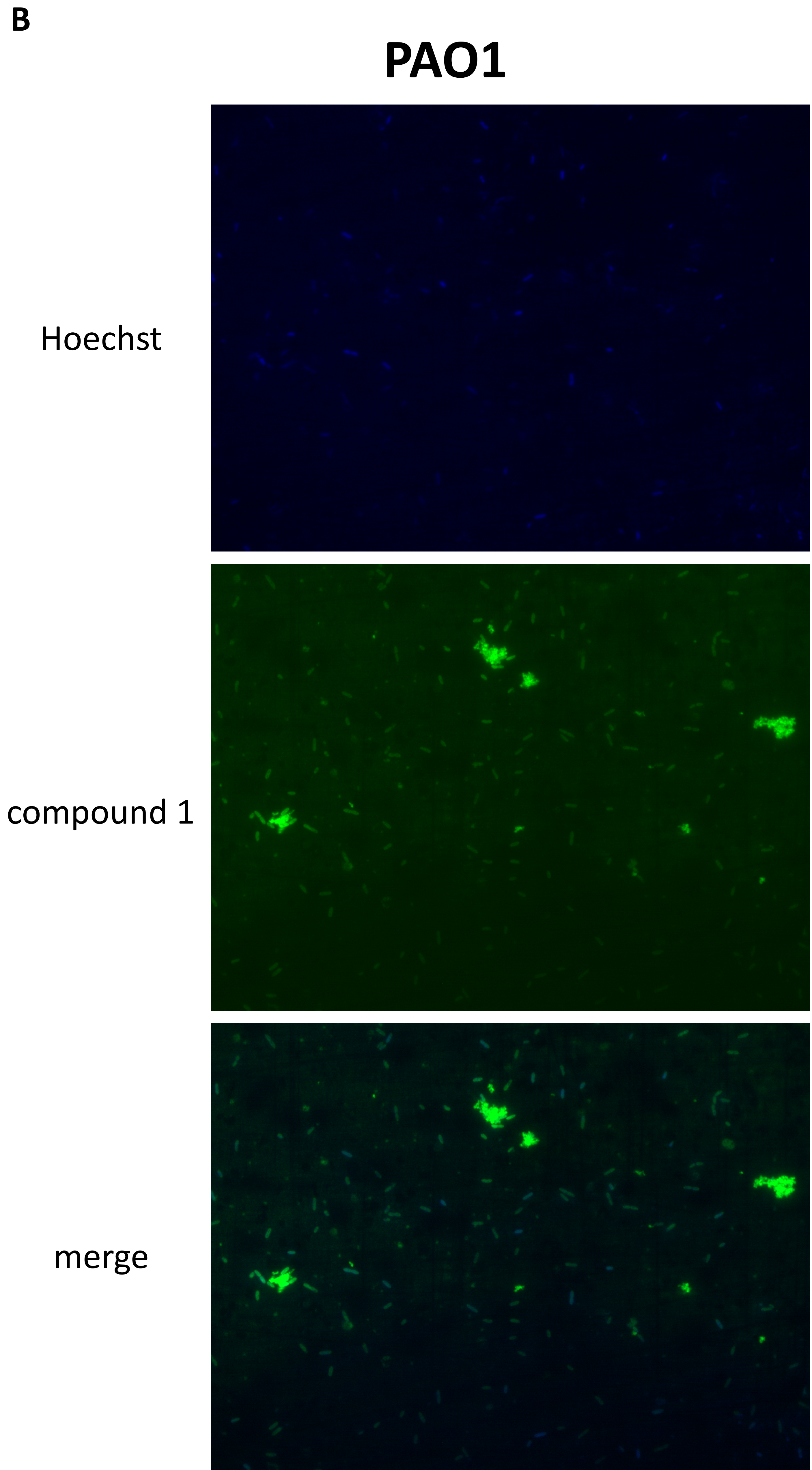
B
PAO1
Hoechst
compound 1
merge

c

STM

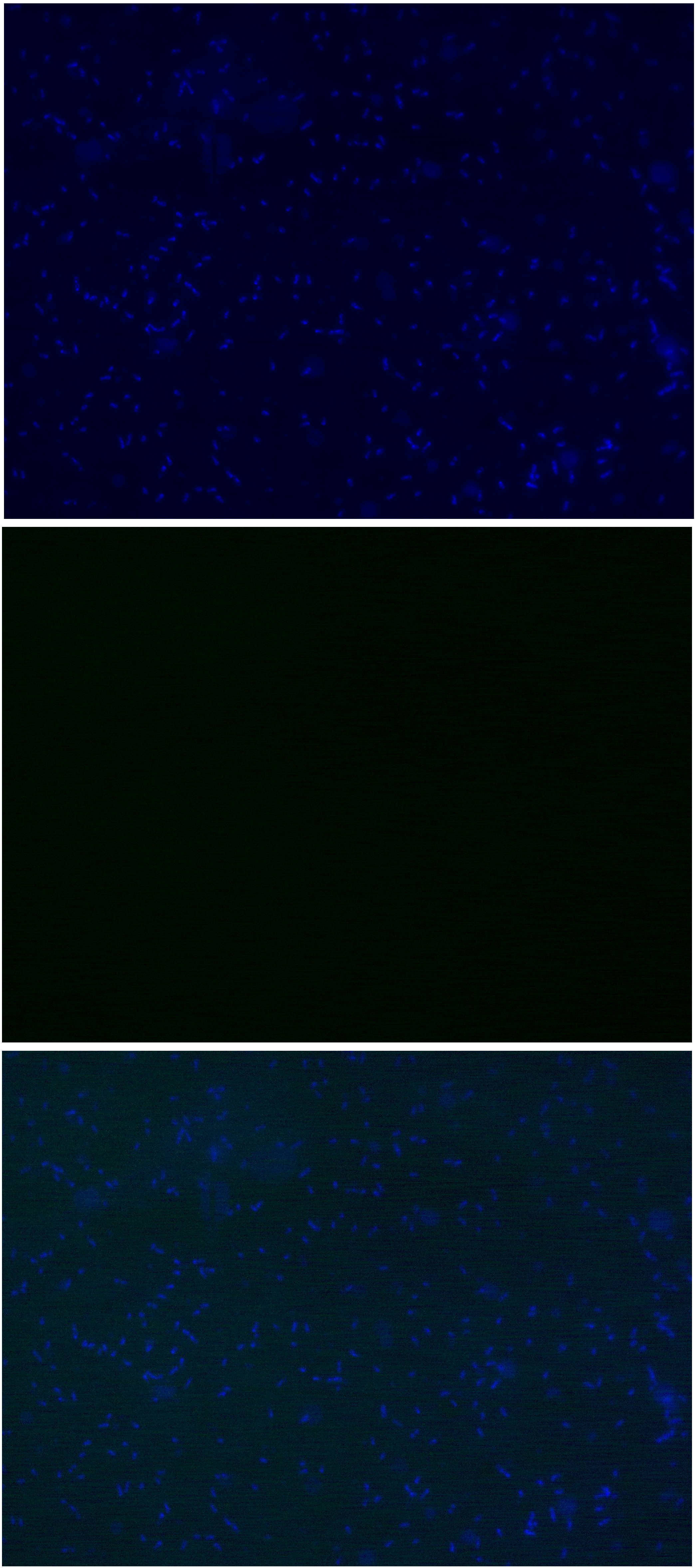

Hoechst

fluo probe

merge

D

PAO1

Hoechst

fluo probe

merge

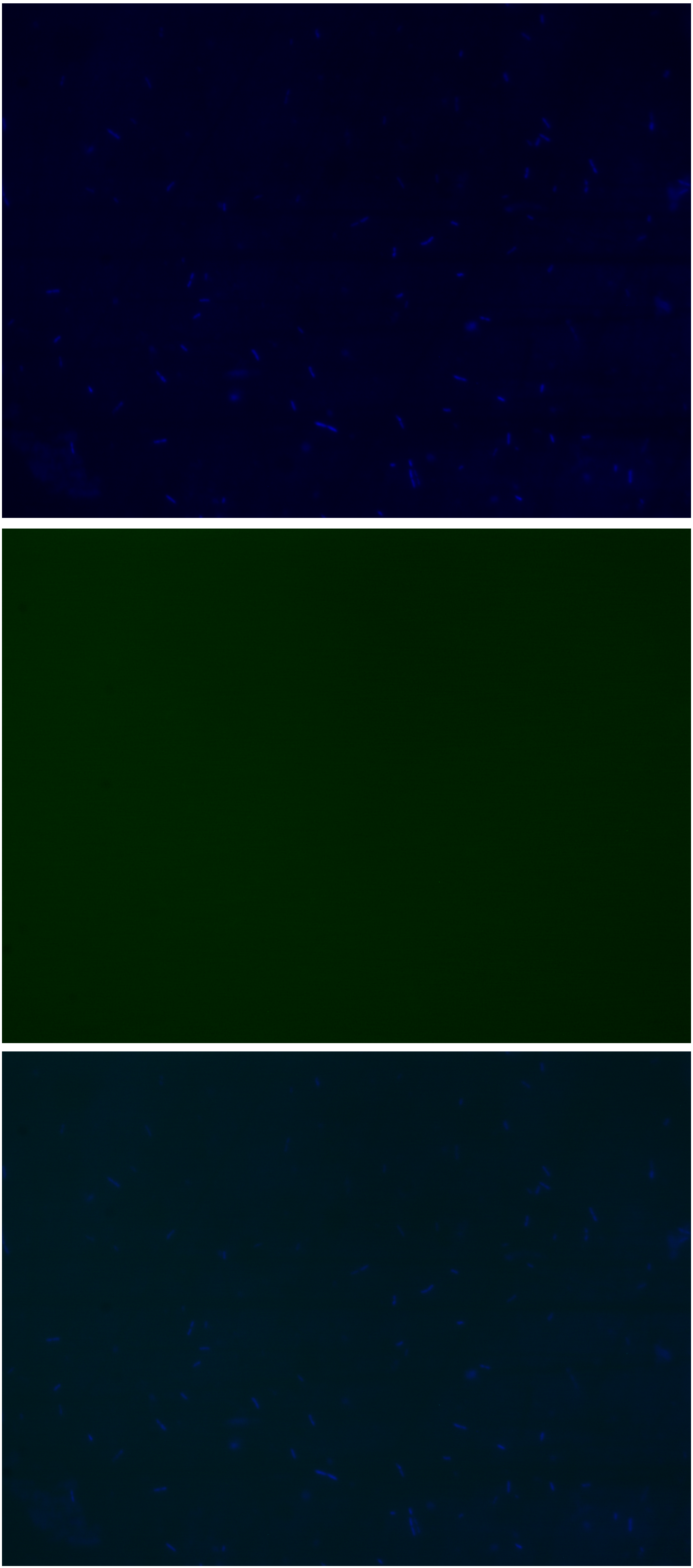

E

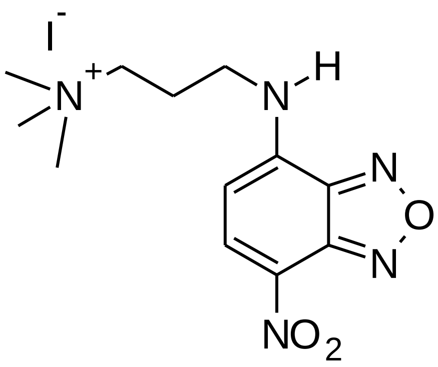

Supplementary Figure S2. Full images of fluorescence microscopy of STM and PAO1 cells after treatment with compound **1** (**panels A** and **B**) or with the fluorescent probe not conjugated to DFP (**panels C** and **D**). As shown in the merge panels, the fluorescent signal from compound **1** does not overlap with any of the STM cells while it merges with PAO1 cells, showing a kind of peripheral distribution in some of them. No signal was detected after incubation of both STM and PAO1 with the fluorescent probe(fluo probe) that lacks the DFP moiety, indicating that the interaction of compound 1 with PAO1 cells is due to DFP. **Panel E**: molecular structure of the fluorescent probe.
